# Serum-Based lncRNA ANRIL, TUG1, UCA1, and HIT Expressions in Breast Cancer Patients

**DOI:** 10.1155/2022/9997212

**Published:** 2022-01-29

**Authors:** Ali G. Alkhathami, Abdul Hadi, Mohammed Alfaifi, Mohammad Yahya Alshahrani, Amit Kumar Verma, Mirza Masroor Ali Beg

**Affiliations:** ^1^Department of Clinical Laboratory Sciences, College of Applied Medical Sciences, King Khalid University, P.O. Box 61413, Abha 9088, Saudi Arabia; ^2^Department of Medicine, Xi'an Jiaotong University, China; ^3^Department of Zoology and Environmental Sciences, GKV, Haridwar, India; ^4^Faculty of Medicine, Alatoo International University, Bishkek, Kyrgyzstan; ^5^Centre for Promotion of Medical Research, Alatoo International University, Bishkek, Kyrgyzstan

## Abstract

Breast cancer is a heterogeneous disease and is the most common and prevalent form of malignancy diagnosed in women. lncRNAs are found to be frequently dysregulated in cancer, and its expression plays a critical role in tumorigenesis. The study included 100 histopathologically confirmed, newly diagnosed untreated patients of invasive ductal carcinoma (IDC) of breast cancer patients and 100 healthy subjects. After blood collection, the serum was separated and total RNA was extracted, cDNA was synthesized using 100 ng of total RNA, and lncRNA (ANRIL, TUG1, UCA1, and HIT) expression was analyzed. Increased ANRIL (3.83-fold), TUG1 (7.64-fold), UCA1 (7.82-fold), and HIT (3.31-fold) expressions were observed in breast cancer patients compared to healthy controls. Relative expression of lncRNAs UCA-1 (*p* = 0.010) and HIT-1 (*p* < 0.0001) was significantly elevated in patients with advanced breast cancer stage compared to those with early-stage disease. While lncRNA TUG-1 expression was found to be higher in patients with early-stage tumors than those with advanced-stage tumors (*p* = 0.06), lncRNA ANRIL showed increased expression in patients with PR positive status (*p* = 0.04). However, we found a significant difference in lncRNA HIT expression in HER-2 positive breast cancer patients compared to HER-2 negative breast cancer patients (*p* = 0.005). An increase in the expression of serum lncRNAs ANRIL (*p* < 0.0001), UCA-1 (*p* = 0.004), and HIT (*p* < 0.0001) was observed in the distant organ metastatic breast cancer patients. In the ROC curve concerning lymph node involvement, the sensitivity and specificity of lncRNA HIT were 68% and 58%, respectively (*p* value = 0.007). In the ROC curve w.r.t. stages of disease, the sensitivity and specificity of lncRNA HIT were 80% and 50%, respectively (*p* value < 0.0001). Better sensitivity and specificity were observed for lncRNA HIT (sensitivity 91% and specificity 78%; *p* value < 0.0001) and ANRIL (sensitivity 70% and specificity 60%; *p* value < 0.0001) w.r.t distant organ metastases.

## 1. Introduction

Breast cancer is a heterogeneous disease characterized by a large number of genetic alterations, and the molecular pathogenesis underlying the disease remains enigmatic and poorly understood [1]. Breast cancer is the most common and prevalent form of malignancy diagnosed in women in the United States and across the globe, and it is the 5th leading cause of cancer-related death after lung cancer among women [2, 3]. Despite remarkable advancements and extensive research made over two decades resulted in early detection and improved the overall prognosis of breast cancer patients, the tumorigenic mechanism of breast cancer remains elusive [4]. Most women with breast cancer patients in stages I to III treated with chemotherapy, hormone therapy, and HER2-targeted drugs, such as trastuzumab (Herceptin) and pertuzumab (Perjeta), followed by surgery and radiotherapy. For stage IV breast cancer patients, systemic (drug) therapies are the main treatments that includes chemotherapy such as anthracycline-based drugs, taxane-based drug, 5-fluorouracil (5-FU), cyclophosphamide, carboplatin, targeted drugs, immunotherapy, and hormone therapy such as tamoxifen, fulvestrant, and aromatase inhibitors. Locally recurrent breast cancer may be treated with breast-conserving surgery (lumpectomy) and mastectomy [5].

The past few decades have witnessed an immense advancement of RNA profiling technologies, and whole-genome transcriptome analysis has helped us to identify a large amount of DNA that is transcribed but does not code for any protein. 75% of the genome is believed to be transcribed into noncoding RNAs, and only 2% of the total genomic sequence is transcribed into protein-coding RNAs in humans [6]. Among the noncoding RNAs, long noncoding RNAs (lncRNAs) are a major subclass and are defined as transcripts more than 200 nucleotides in length, containing no open reading frame, and hence, having no protein-coding capacity [7]. lncRNAs are found to regulate many important physiological and biological processes including cell differentiation, genome packaging, apoptosis, metabolism, and gene regulation [8, 9]. Evidence has indicated that dysregulated lncRNA expression plays a role in tumorigenesis. lncRNAs are often found dysregulated in carcinomas, and hence, they are assumed to be a critical component of cancer biology, controlling progression, recurrence, metastasis, and poor prognosis [10–12].

7.1 kb long lncRNA taurine-upregulated gene 1 (TUG-1) is located on chromosome 22q12. TUG-1 is aberrantly expressed in cancer and has been involved in the progression of various types of tumors: hepatocellular carcinoma, non-small-cell lung cancer (NSCLC), cancer of the bladder, and cancer of the colon [13–15]. Recent studies have shown an association between the augmented expression of TUG-1 and clinical variables in breast cancer tissues [16, 17]. However, the exact molecular control mechanism of TUG1 in breast cancer progression remains unclear. In the cytoplasm of TNBC tissues and cells that have been shown to support in vivo and in vitro invasion, EMT, and metastasis experiments, Negatively induced androgen receptor lncRNA (ANRIL) is located [18]. Accumulating evidence indicates that ANRIL is a new and emerging prognostic biomarker for triple-negative breast cancer (TNBC), but the underlying mechanisms are still unknown.

Urothelial cancer-associated 1 (UCA1), with a 1.4 kb long transcript, is a long noncoding RNA (lncRNA) that was identified in a bladder cancer cell line [19]. Several studies have reported the oncogenic roles of this lncRNA in different tissues, i.e., breast cancer, colorectal cancer, gastric cancer, ovarian cancer, and hepatocellular carcinoma [20]. A recent study has subsequently reported that UCA1 can upregulate protein tyrosine phosphatase 1B (PTP1B) to increase cell proliferation in breast cancer, providing new insights into the mechanism of UCA1 regulation of breast cancer, which could be a potential target for the treatment of breast cancer [21]. lncRNA-HIT (transforming growth factor- (TGF-) *β*-induced HOXA transcript) has recently been identified and shown to play an important role in the transition from epithelial to mesenchymal and cell growth [22, 23]. However, the potential role of lncRNA HIT in breast cancer growth and progression remains unclear, and little is known about its oncogenic properties and molecular mechanisms in breast cancer cells.

In this study, we aimed to determine the expression of ANRIL, TUG1, UCA1, and HIT lncRNAs in breast cancer patients. We investigated the differential expression of four pairs of lncRNA and analyzed their oncogenic roles and associations with clinical and pathological features. Furthermore, we also investigated the potential correlations between circulating lncRNAs and patient clinicopathological factors.

## 2. Materials and Methods

### 2.1. Subject Recruitment and Sample Collection

Histopathologically confirmed 100 untreated patients of invasive ductal carcinoma (IDC) of breast and 100 healthy subjects were included in the current study. Peripheral blood sample was withdrawn from all the recruited patients and healthy controls in plain vials after informed consent obtained. Withdrawn sample was centrifuged at 1500 rpm to collect the serum and stored at -80°C for further processing. The institutional ethical committee Gurukula Kangri University, Haridwar, India ethically approved this research study (27/07/2017/GKV/IEC/2017), and informed written consent was obtained from all the participants before the study commenced at the Department of Zoology, Gurukula Kangri University, Haridwar, India.

### 2.2. Total RNA Extraction

Total RNA extraction from the stored serum was performed from serum, and the serum was taken out, thawed, and processed for total RNA extraction using TRIzol following the manufacturer's protocol. The purity and quality of RNA were determined by the A260/280 ratio using a spectrophotometer and stored at -80°C until an additional necessary step for cDNA synthesis.

### 2.3. Complementary DNA Synthesis and QRT PCR

Complementary DNA was synthesized using a kit (Verso, Thermo Scientific, USA) following the manufacturer protocol; 100 ng of total extracted serum RNA was converted into cDNA. Serum-based noncoding RNA INK4 locus (ANRIL), taurine-upregulated gene 1 (TUG1), urothelial cancer-associated 1 (UCA1), and HOXA TGF*β*-induced transcript (HIT) expression was determined by quantitative real-time PCR using SYBR Green dye with specific primer sequences in QRT-PCR (Table S1). The following program was used for qRT-PCR to amplify the lncRNAs ANRIL, TUG1, UCA1, and HIT and GAPDH in a 20 *μ*l reaction volume: 40 cycles of initial denaturation at 94°C for 40 seconds; annealing for 40 seconds at 60°C for lncRNAs (ANRIL, TUG1, UCA1, HIT), and GAPDH; and extension at 72°C for 40 seconds. To end the reaction, a final step was used at 72° C for 5 minutes and a melting curve test was performed between 35° C and 90° C to confirm target amplification. Each experiment included a control sample without cDNA, and every reaction was carried out in duplicate. Relative quantification by the 2^−(*ΔΔ*CT)^ method was used to compute expression levels of the lncRNAs ANRIL, TUG1, UCA1, and HIT.

### 2.4. Statistical Analysis

All the data interpretation and computation were performed using GraphPad Prism software, version 6.05 and SPSS 21.0. 2-group comparison was done by Mann–Whitney *U* test to check the significance of differences. Fold change in expression of the qRT-PCR results was performed with the relative cycle threshold (Ct) method. lncRNAs ANRIL, TUG1, UCA1, and HIT expression levels were calculated by the relative quantification method using 2^–(*ΔΔ*Ct)^. Genes whose expression was increased or decreased by more than one-fold were considered to be upregulated or downregulated, respectively. All values were normalized relative to the control values, which were set at 1. A *p* value of less than 0.05 indicated significance.

## 3. Results

### 3.1. General Characteristics of the Study Population

The clinicopathological characteristics of the breast cancer group and healthy control group are shown in detail (Table 1). In brief, a total of 100 patients with invasive ductal carcinoma of the breast and 100 healthy controls were included in the study. Additionally, subtype distribution and key molecular data types for clinically confirmed patients of breast cancer patients and corresponding age-matched healthy controls were evaluated; all the participants were female (Table 1) [24].

### 3.2. Circulating Serum lncRNA Relative Expression Profiles with respect to Clinicopathological Features

**Figure 1:**
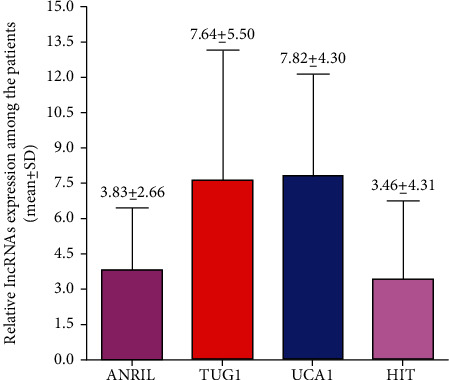
Relative expression of ANRIL, TUG1, UCA1, and HIT lncRNAs among the breast cancer patients.

We analyzed the relative expression levels of different circulating lncRNAs in each serum sample of breast cancer patients. The relative expression of different lncRNAs studied such as ANRIL (3.83 fold), TUG-1 (7.64 fold), UCA-1 (7.82 fold), and HIT (3.46 fold) (Figure 1) and their association with clinical and pathological characteristics among the breast cancer patients (Tables 2–5) was obtained. ANRIL expression was found to be higher in the advanced-stage tumors than in the early-stage tumors (4.18 ± 2.41 vs. 3.26 ± 2.12; *p* = 0.12, Table 2), though the difference was not statistically significant. Similarly, the relative expression of lncRNAs UCA-1 (8.37 ± 4.14 vs. 6.91 ± 4.45; *p* = 0.010) and HIT-1 (4.41 ± 3.97 vs. 1.51 ± 0.90; *p* < 0.0001) was significantly elevated in patients with advanced breast cancer stage than in those with early-stage tumors (Tables 4 and 5). Contrary to the abovementioned findings, lncRNA TUG-1 expression was found to be higher in patients with early-stage tumors than in those with advanced-stage tumors (8.45 ± 8.00 vs. 7.42 ± 2.41; *p* = 0.06); however, the differences was not statistically significant (Table 3).

### 3.3. Circulating Serum lncRNA Relative Expression Profiles with respect to ER, PR, HER-2, and Distant Metastasis Status

Prior studies have suggested that ER and PR are involved in the development of mammary glands and play a critical role in the development and progression of breast cancer [25]. We divided patients into ER/PR positive and ER/PR negative groups and observed whether there was any difference in the serum lncRNAs studied. Our results showed that out of all the studied lncRNAs, only ANRIL had increased expression in patients with PR positive status (4.89 ± 3.52; *p* = 0.04) (Table 2), suggesting that lncRNA ANRIL may be associated with female PR levels. Second, we also investigated the difference of circulating serum lncRNAs between HER-positive and HER-negative patients. We could not find any significant difference in the expression levels of lncRNAs ANRIL, TUG-1, and UCA-1 with respect to HER-2 status, but we did observe an increasing tendency of these lncRNAs in HER-2 positive patients. However, we found a significant difference in expression levels of lncRNA HIT in HER-2-positive breast cancer patients compared to HER-2-negative breast cancer patients (4.23 ± 4.02 vs. 2.92 ± 2.70; *p* = 0.005) (Table 5). We further compared the serum lncRNA expression levels based on the distant metastases of the tumor in breast cancer patients. We found a significant increase in the expression of serum lncRNAs ANRIL (5.69 ± 2.78; *p* < 0.0001), UCA-1 (9.26 ± 4.58; *p* = 0.004), and HIT (7.37 ± 4.38; *p* < 0.0001) in the distant metastases positive breast cancer patients. No significant difference was observed in the expression of lncRNAs with respect to the involvement of lymph nodes and menopausal status (Tables 2–5; Figure 1).

### 3.4. Prognostic Value of lncRNAs (ANRIL, TUG-1, UCA-1, and HIT) with respect to Clinicopathological Parameters

**Figure 2:**
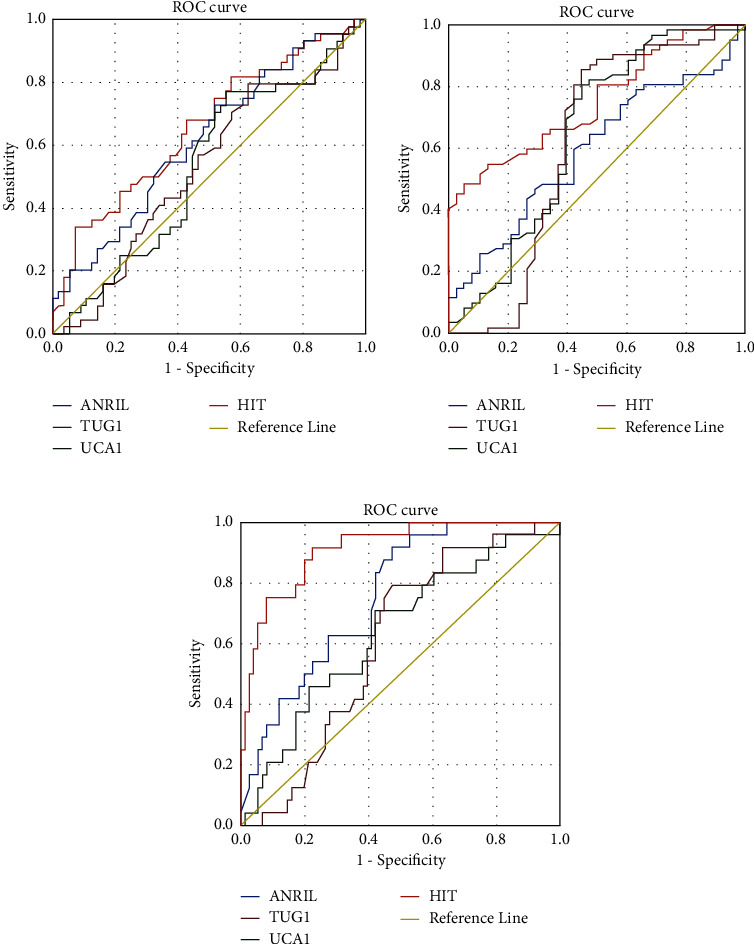
ROC curve for lncANRIL, AUG1, UCA1, and HIT expressions (a) with respect to lymph node involvement and noninvolvement, (b) with respect to the early and advanced stages of disease, and (c) with respect to distant organ metastases and without metastases.

Receiver operating characteristic (ROC) curves were used to test the feasibility of using circulating serum lncRNAs as a prognostic marker for breast cancer detection. Additionally, to determine and better understand the potential value of these lncRNAs as the most accurate clinical biomarker for breast cancer screening, we compared the sensitivity and specificity of lncRNAs with respect to different clinicopathological parameters (Figure 2). The ROC curve and area under the ROC curve (AUC) for the lncRNAs ANRIL, TUG-1, UCA-1, and HIT with respect to different clinicopathologic variables are indicated in Figures 2(a)–2(c). With respect to involvement and noninvolvement of lymph nodes, the ROC and AUC for the studied lncRNAs were analyzed. We found the sensitivity and specificity of lncRNA HIT to be 68% and 58%, respectively (*p* value = 0.007 and AUC of 0.65). The sensitivity of lncRNA ANRIL was 65%, with a specificity of 52% (*p* value = 0.04 and AUC of 0.61). The sensitivity of TUG-1 and UCA-1 was 56% and 61%, the specificity was 52% and 54%, and the AUC was 0.52 and 0.53, respectively (Figure 2(a), Table 6). Among the lncRNAs studied, HIT demonstrated the best sensitivity and specificity with respect to the involvement or noninvolvement of lymph nodes, confirming that the lncRNA HIT may serve as a better biomarker to increase the detection rate and may have a better diagnostic value than the other circulating lncRNAs in the serum sample of breast cancer patients.

Furthermore, we also determined the ROC for the abovementioned lncRNAs with respect to the early and advanced stages of the disease. We found the sensitivity and specificity of lncRNA HIT to be 80% and 50%, respectively (*p* value < 0.0001 and AUC of 0.75). The sensitivity of the lncRNA ANRIL was 64%, and its specificity was 53% (*p* value = 0.06 and AUC of 0.59). The sensitivity of TUG-1 and UCA-1 was 72% and 71%, the specificity was 61% and 58%, and the AUC was 0.61 and 0.64, respectively (Figure 2(b), Table 6).

Given the clinical importance of metastasis in breast cancer, identifying novel lncRNAs related to metastatic potential of breast cancer is very important. Therefore, we also performed ROC analysis, which showed a better sensitivity and specificity for lncRNA HIT (sensitivity 91% and specificity 78%; AUC = 0.91; *p* value < 0.0001) and ANRIL (sensitivity 70% and specificity 60%; AUC = 0.75; *p* value<0.0001) compared to other studied lncRNAs with respect to distant organ metastases and without metastases (Figure 2(c), Table 6). Overall, in the current study, we found lncRNA HIT to be a better diagnostic and prognostic indicator for breast cancer with respect to various established clinicopathologic variables, which may hold a clinical significance and add great value to breast cancer diagnosis.

### 3.5. Survival Analysis

**Figure 3:**
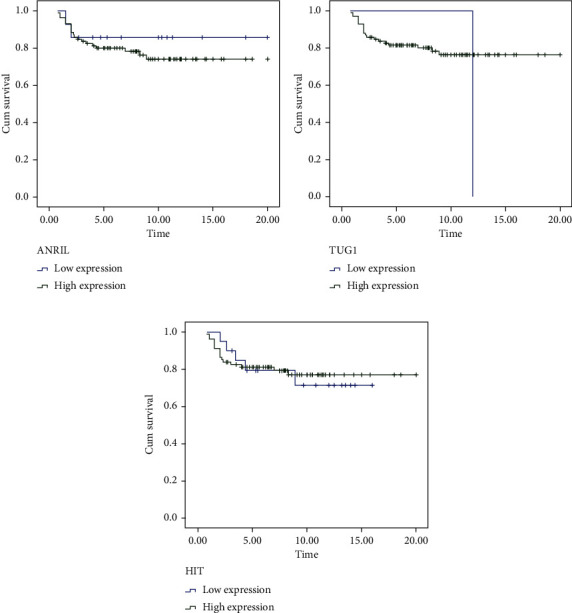
Survival curve for breast cancer patients with respect to high and low expression: (a) ANRIL expression, (b) TUG1 expression, and (c) HIT expression.

Survival analysis of breast cancer patients were performed based on low (≤1 fold) or high (>1 fold) expression of lnc ANRIL, TUG1, UCA1, and HIT RNAs (Figure 3). We observed that the breast cancer patients have low and high expression in ANRIL, TUG1, and HIT lncRNAs and none patients who had low expression in UCA1. Since the survival were evaluated for ANRIL, TUG1, and HIT RNAs among the breast cancer patients. Patients with low expression and high expression of lncRNA ANRIL had 17.39 months and 14.62 months of median survival, respectively (*p* = 0.49). Patients who had low expression of lncRNA TUG1 had 12.00 months of median survival while patients with high expression had 16.0 of median survival (*p* = 0.20). Patients with low expression of lncRNA HIT expression showed 16.08 months of median survival while high expression of lncRNA HIT in patients showed 13.08 months of median survival (*p* = 0.96). Overall, if we see the impact of lncRNA expression with relation to survival of patients, no such significant association was observed.

## 4. Discussion

Breast cancer remains a leading cause of cancer-related deaths among females worldwide [1], and although significant progress has been made to improve clinical management to reduce the mortality rate, a substantial number of patients still face cancer recurrence [2, 3, 5]. Accumulating evidence has revealed that lncRNAs play an important role in cancer development and progression. Furthermore, it has been established that breast cancer is a complex and heterogeneous disease and involves in the interplay of multiple factors. Therefore, traditional prognostic factors, including TNM stage, tumor grade, and lymph node status, do not predict the risk of tumor progression and overall survival in breast cancer patients [8, 9]. The past few decades have witnessed an immense advancement in RNA profiling technologies and whole-genome transcriptome analysis that have helped to decipher the role of lncRNAs as key regulators of cancer development and progression [10–12]. However, the expression profile and prognostic significance of many lncRNAs in breast cancer have not been clinicopathologically compared and systematically explored. Additionally, the number of well-characterized lncRNAs as a potential biomarker for disease progression and prognosis in breast cancer appears to be quite rare.

In the current study, we analyzed the expression profiles of TUG1, ANRIL, UCA1, and HIT lncRNAs in breast cancer patients and investigated the differential expression of four pairs of lncRNA to determine their oncogenic roles and associations with clinical and pathological features. We envisioned that multidimensional evaluation by means of analyzing a panel of established blood biomarkers might be more accurate in predicting the development and progression of breast cancer than predictors based on any single biomarker or clinical variables alone. In the present study, we revealed a critical role of lncRNA-HIT in breast cancer patients. Out of the studied biomarkers, the predictive value and robustness of lncRNA-HIT was found to be more significantly correlated with the various clinicopathological variables studied.

We found a significant difference in the expression levels of the lncRNA HIT in HER-2-positive breast cancer patients compared to HER-2-negative breast cancer patients (*p* = 0.005). We analyzed the circulating serum lncRNAs as a diagnostic and prognostic marker for the detection of breast cancer by plotting receiver operating characteristic (ROC) curves. We compared the sensitivity and specificity of lncRNAs with respect to different clinicopathological parameters. With respect to involvement and noninvolvement of lymph nodes, we found the sensitivity and specificity of lncRNA-HIT to be 68% and 58%, respectively (*p* value = 0.007). The sensitivity of lncRNA ANRIL was 65%, and its specificity was 52% (*p* value = 0.04). We also observed a significant increase in the expression of serum lncRNAs ANRIL and HIT (*p* < 0.0001) in the distant metastasis-positive breast cancer patients, suggesting that these lncRNAs may have clinical significance in breast cancer diagnosis and prognosis.

Additionally, ROC analysis revealed a better sensitivity and specificity of lncRNA HIT (*p* value < 0.0001) with respect to the early and advanced stages of the disease and with respect to distant organ metastases and without metastases (*p* value < 0.0001), and this was followed by ANRIL (*p* value < 0.0001), compared to the other lncRNAs studied.

Our results can be explained by the fact that lncRNA-HIT expression has been found to be significantly elevated in the highly metastatic cell line 4T1 compared to isogenic mouse cell lines with less metastatic capacity [22, 26]. Furthermore, in the current study, we showed that lncRNA-HIT demonstrated a better predictive capacity with respect to clinicopathological factors including tumor grade, HER, and lymph node status. In fact, HIT was the top upregulated lncRNA shown in our study, and it has been shown to play an important role in TGF-ß induced EM, cell migration, and invasion [22, 23] Thus, the expression of human lncRNA-HIT is associated with more invasive tumor and breast cancer development and progression. lncRNA-HIT was significantly upregulated in NSCLC tissues and cell lines, and the expression level of lncRNA-HIT involved in to induced epithelial-to-mesenchymal transition and cell growth [22] as well as involved in cell migration and invasion [22].

It has been shown to be upregulated in hepatocellular carcinoma and act as an oncogene through association with RNA binding protein E2F1 in NSCLC cells, suggesting that this lncRNA-HIT may exert its biological function by functionally associating with transcription factors [21]. Additionally, a recent study has reported that lncRNA-HIT is involved in cell migration and invasion through its association with another transcription factor, ZEB-1 [27]. Overall, out of the biomarkers studied, the predictive value and robustness of lncRNA-HIT was found to be more significantly correlated with the various clinicopathological variables studied. Thus, it is a plausible inference that the lncRNA HIT may be associated with breast cancer-related biological processes and pathways, and its dysregulated expression may contribute to the development and progression of breast cancer in humans. Hence, taken together, the broad and wide array of stimuli, including association with the clinicopathological variables of breast cancer, serum levels of the lncRNAs ANRIL, and more precisely, HIT could serve as an “early” surrogate of disease progression and/or activity that may signal an increased risk of a poor outcome. Finally, although we have shown a link between lncRNA HIT and the progression of disease, we cannot infer its pathological role or differentiate between the progression rates of each of the associated conditions. Specific mechanistic studies in breast cancer tissue and intervention studies evaluating the benefit of therapeutic approaches stratified by biomarker levels are therefore urgently justified in clarifying the usefulness in clinical practice of this prognostic biomarker. Additionally, many computational models have been developed that are effective in predicting lncRNA-disease associations [28]. Several studies were done to state that on lncRNAs of the present study could be the potential prognostic marker. Wang et al.'s study suggested that lncRNA ANRIL overexpression was significantly associated with lymph node metastases, TNM stage, and poor prognosis and revealed that lncRNA ANRIL may serve as a novel biomarker for lymph node metastases and disease prediction. [29]. Higher ANRIL expression was linked with poor clinical outcome and could be the unique potential prognostic biomarker [30]. Higher lncRNA TUG1 expression appears to be predictive of distant metastases and advanced tumor stage and could serve as a biomarker for poor prognosis in cancers [31]; lncRNA TUG1 may stimulate the development of osteosarcoma, which can serve as a prognosis and diagnostic marker [32]. Upregulated UCA1 expression was observed in patients with gastric cancer and suggested that its upregulation could regulate the spread of gastric cancer cells and metastases [33] and significantly associated with lymph node metastases and the TNM stage could be the biomarker of malignancy [34]. The expression of *lncRNA-HIT* was significantly upregulated in NSCLC and cell lines, and higher expression of *lncRNA-HIT* correlates with advancement of disease stage and predicts unfavorable prognosis of NSCLC patients may serve as novel valuable marker for the prognosis of NSCLC [35].

In conclusion, our clinical data show that lncRNA-HIT is upregulated in serum samples of breast cancer patients and indicates that lncRNA expression alteration could be the prognostic biomarker for prediction of condition of disease. Thus, this biomarker panel may enable the determination of the severity and course of disease progression. The current study not only indicated the potential role of lncRNA-HIT as a candidate biomarker beyond the established clinical prognostic factors in breast cancer patients but also help increase our understanding of the molecular machinery underlying breast cancer development and progression with further prospective validation. However, our study had certain limitations such as the study was performed on lncRNAs using patient serum samples. We would like to perform the same analyses in the tissue samples of the patients. Second, our study did not have an independent cohort to identify the predictive value of the studied biomarkers.

## Figures and Tables

**Table 1:** Demographic and clinical characteristic of breast cancer patients [24].

Variables	Breast cancer patients 100 (%)	Healthy controls 100 (%)
*Age*		
≤50 years	54 (54)	60 (60)
>50 years	46 (46)	40 (40)
*Menopause*		
Yes	67 (67)	64 (64)
No	33 (33)	36 (36)
*Lymph nodes*		
Yes	44 (44)	
No	56 (56)	
*ER status*		
Yes	31 (31)	
No	69 (69)	
*PR status*		
Yes	28 (28)	
No	72 (72)	
*Her2 status*		
Yes	40 (40)	
No	60 (60)	
*TNM stages*		
Early stage (I & II)	38 (38)	
Advanced stage (III & IV)	62 (62)	
*Distant metastases*		
Yes	24 (24)	
No	76 (76)	

**Table 2:** Long noncoding RNA-ARNILA expression and clinicopathological features.

Variables	Long noncoding RNA-ANRIL		*p* value
	Mean	SD	
*Relative expression*	3.83	2.66	—
*Age*			
≤50 years	3.90	2.95	0.90
>50 years	3.75	2.30	
*Menopause*			
Yes	3.24	2.51	0.10
No	4.12	2.70	
*Lymph nodes*			
Yes	3.31	2.31	0.04
No	4.50	2.94	
*ER status*			
Yes	4.02	2.79	0.69
No	3.74	2.61	
*PR status*			
Yes	4.89	3.52	0.04
No	3.42	2.13	
*Her2 status*			
Yes	3.89	3.80	0.98
No	2.87	2.53	
*TNM stages*			
Early stage (I&II)	3.26	2.12	0.12
Advanced stage (III&IV)	4.18	2.90	
*Distant metastases*			
Yes	5.69	2.78	<0.0001
No	3.25	2.35	

**Table 3:** Long noncoding RNA-TUG1 expression w.r.t clinicopathological features.

Variables	Long noncoding RNA-TUG1		*p* value
	Mean	SD	
*Relative expression*	7.64	5.50	—
*Age*			
≤50 years	8.01	7.06	0.58
>50 years	7.21	2.74	
*Menopause*			
Yes	7.83	5.84	0.55
No	7.27	4.82	
*Lymph nodes*			
Yes	7.28	3.21	0.62
No	7.93	6.80	
*ER status*			
Yes	7.01	2.89	0.95
No	7.93	6.34	
*PR status*			
Yes	6.95	2.42	0.93
No	7.91	6.30	
*Her2 status*			
Yes	8.04	4.46	0.26
No	7.38	6.12	
*TNM stages*			
Early stage (I & II)	8.45	8.00	0.06
Advanced stage (III&IV)	7.42	2.41	
*Distant metastases*			
Yes	7.86	2.41	0.12
No	7.58	6.18	

**Table 4:** Long noncoding RNA-UCA1 and long noncoding RNA-TUG1.

Variables	Long noncoding RNA-UCA1		*p* value
	Mean	SD	
*Relative expression*	7.82	4.30	—
*Age*			
≤50 years	7.96	4.46	0.90
>50 years	7.46	4.14	
*Menopause*			
Yes	7.89	4.42	0.95
No	7.66	4.10	
*Lymph nodes*			
Yes	7.83	4.05	0.58
No	7.80	4.52	
*ER status*			
Yes	7.86	4.00	0.79
No	7.80	4.45	
*PR status*			
Yes	8.08	3.94	0.39
No	7.71	4.45	
*Her2 status*			
Yes	8.48	4.20	0.09
No	7.37	4.34	
*TNM stages*			
Early stage (I & II)	6.91	4.45	0.01
Advanced stage (III & IV)	8.37	4.14	
*Distant metastases*			
Yes	9.26	4.58	0.04
No	7.36	4.13	

**Table 5:** Long noncoding RNA-HIT and long noncoding RNA- UCA1.

Variables	Long noncoding RNA-HIT		*p* value
	Mean	SD	
*Relative expression*	3.46	3.31	—
*Age*			
≤50 years	3.15	3.0	0.44
>50 years	3.51	3.20	
*Menopause*			
Yes	3.51	3.59	0.22
No	2.90	3.20	
*Lymph nodes*			
Yes	4.42	4.19	0.007
No	2.44	2.48	
*ER status*			
Yes	3.06	2.89	0.96
No	3.71	3.43	
*PR status*			
Yes	3.64	3.29	0.26
No	3.54	3.19	
*Her2 status*			
Yes	4.23	4.02	0.05
No	2.92	2.70	
*TNM stages*			
Early stage (I & II)	1.51	0.90	<0.0001
Advanced stage (III & IV)	4.41	3.97	
*Distant metastases*			
Yes	7.37	4.38	<0.0001
No	2.03	1.74	

**Table 6:** AUC curve for lncRNA ANRIL, TUG1, UCA1, and HIT with respect to different categories (lymph node involvement vs. noninvolvement, early vs. advanced stages of disease, distant organ metastases vs. without metastases).

With respect to lymph node involvement and noninvolvement					
lncRNAs	AUC (95% CI)	Cut off	Sensitivity	Specificity	*p* value
ANRIL	0.61 (0.50-0.72)	3.24	65%	52%	0.04
TUG1	0.52 (0.41-0.64)	6.79	56%	52%	0.62
UCA1	0.53 (0.40-0.63)	6.38	61%	54%	0.58
HIT	0.65 (0.55-0.76)	1.74	68%	58%	0.007
With respect to the early and advanced stages of disease					
lncRNAs	AUC (95% CI)	Cut off	Sensitivity	Specificity	*p* value
ANRIL	0.59 (0.48-0.70)	3.00	64%	53%	0.12
TUG1	0.61 (0.47-0.74)	5.93	72%	61%	0.06
UCA1	0.64 (0.52-0.76)	5.83	71%	58%	0.01
HIT	0.75 (0.65-0.84)	1.32	80%	50%	<0.0001
With respect to T2DM patients with respect to distant organ metastases and without metastases					
lncRNAs	AUC (95% CI)	Cut off	Sensitivity	Specificity	*p* value
ANRIL	0.75 (0.65-0.85)	3.63	70%	60%	<0.0001
TUG1	0.60 (0.48-0.71)	7.00	70%	57%	0.12
UCA1	0.63 (0.51-0.76)	6.49	70%	58%	0.04
HIT	0.91 (0.85-0.97)	2.43	91%	78%	<0.0001

## Data Availability

The datasets used and/or analyzed during the present study are available from the corresponding author.

## Supplementary Materials

Supplementary Materials Table S1: primer sequences for amplification of lncRNA ANRIL, TUG1, UCA1, and HIT.
