# Supplementary material for: Serum-Based lncRNA ANRIL, TUG1, UCA1, and HIT Expressions in Breast Cancer Patients
Source: Dis Markers. 2022 Jan 29;2022:9997212. doi: 10.1155/2022/9997212 (PMC8817891; doi:10.1155/2022/9997212)
Supplement: Supplementary Materials — Table S1: primer sequences for amplification of lncRNA ANRIL, TUG1, UCA1, and HIT. [file 9997212.f1.pdf]

Table S1: Primer sequences for amplification of lncRNA ANRIL, TUG1, UCA1 and HIT.

Primer sequences	Annealing temperature
**ANRIL**<br>Forward, 5'-GCCGGACTAGGACTATTTGCC-3'<br>Reverse, 5'-TGGCACATACCACACCCTAAC-3'	60°C
**TUG1**<br>Forward, 5'- CGACTGAGCAAGCACTACCA -3'<br>Forward, 5'- CTCAGCAATCAGGAGGCACA -3'	60°C
**UCA1**<br>Forward, 5'- ATTAGGCCGAGAGCCGATCA -3'<br>Forward, 5'- CCAGAGGAACGGATGAAGCC-3'	60°C
**HIT**<br>Forward,<br>5'-TGAAAGGGAGAGAAAGGAAAGG-3'<br>Forward, 5'- -<br>GACAGTCTAGGCATTGCTGAT-3'	60°C
**GAPDH**<br>Forward, 5'-GGTGGTCTCCTCTGACTTCAA -3'<br>Reverse, 5'-GTTGCTGTAGCCAAATTCGTTGT<br>-3'	60°C
